# Supplementary material for: Volatile and non-volatile pathogen cues shape host extracellular vesicles production in pre-infection response
Source: Nat Commun. 2025 Dec 21;17:1038. doi: 10.1038/s41467-025-67789-z (PMC12847967; doi:10.1038/s41467-025-67789-z)
Supplement: Supplementary file 1 — Supplementary Information [file 41467_2025_67789_MOESM1_ESM.pdf]

Supplementary Information

Volatile and non-volatile pathogen cues shape host extracellular vesicles production in pre-infection response

Klaudia Kołodziejska[1,2], Agata Szczepańska[1], Satya Vadlamani[1,2#], Ramakrishnan Ponath Sukumaran[1,2#], Mariusz Radkiewicz[3], Henrik Bringmann[4], Nathalie Pujol[5], Wojciech Pokrzywa[6], Michał Turek[1]

Affiliations:

[1]*Laboratory of Animal Molecular Physiology, Institute of Biochemistry and Biophysics, Polish Academy of Sciences, Warsaw, Poland*

[2]*Doctoral School of Molecular Biology and Biological Chemistry at Institute of Biochemistry and Biophysics, Polish Academy of Sciences, Warsaw, Poland*

[3]*Mass Spectrometry Facility, Institute of Biochemistry and Biophysics, Polish Academy of Sciences, Warsaw, Poland*

[4]*Biotechnology Center, Center for Molecular and Cellular Bioengineering, Technische Universität Dresden, Dresden, Germany*

[5]*Aix Marseille Univ, INSERM, CNRS, CIML, Turing Centre for Living Systems, 163 Avenue de Luminy, case 906, 13009 Marseille, France*

[6]*Laboratory of Protein Metabolism, International Institute of Molecular and Cell Biology in Warsaw, Poland*

[#] These authors contributed equally

Correspondence should be directed to:

MT: m.turek@ibb.waw.pl

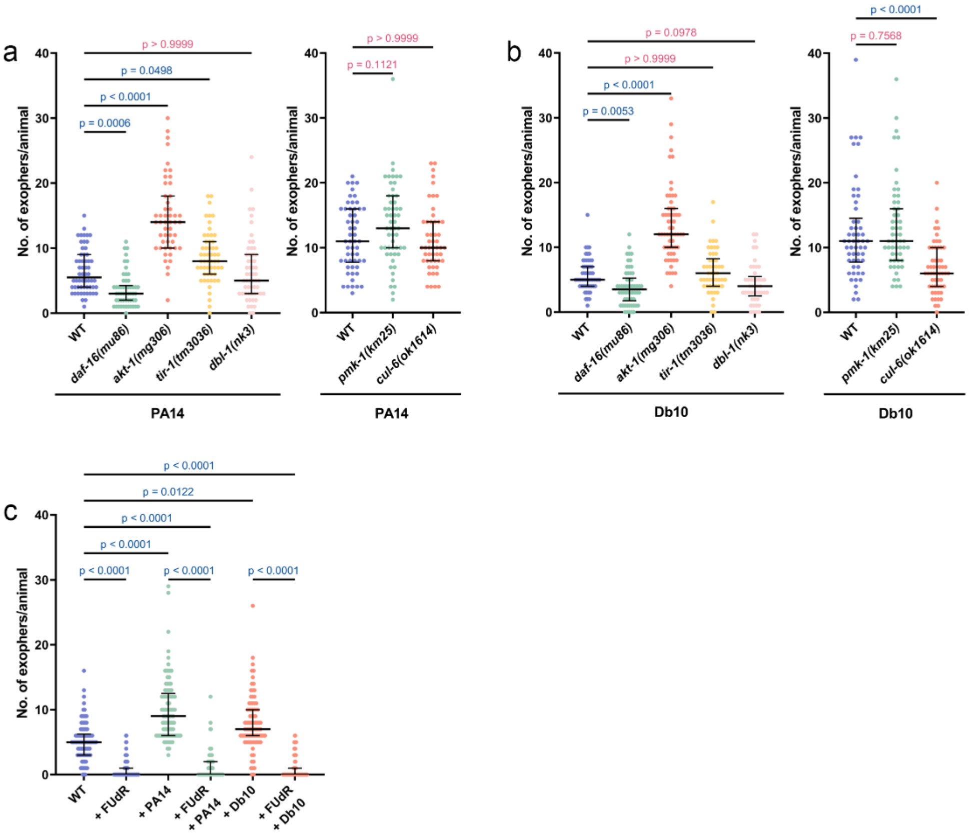

Supplementary Figure 1. Worms deficient in key immune signalling pathways exhibit altered exopher production in response to pathogenic bacterial exposure.

a Exopher production in immune signaling mutants following exposure to PA14 (n = 58, 54, 50, 55, and 56 worms (for respective columns), N = 2 independent experiments; n = 54, 55, and 51 worms (for respective columns), N = 2 independent experiments).

b Exopher production in immune signaling mutants following exposure to Db10 (n = 59, 58, 60, 50, and 45 worms (for respective columns), N = 2 independent experiments; n = 54, 55, and 56 worms (for respective columns), N = 2 independent experiments).

c Treatment with FUdR significantly reduced exopher production in worms, regardless of exposure to PA14 or Db10 (n = 90, 90, 89, 69, 85, and 85 worms (for respective columns), N = 3 independent experiments).

Data information: Differences in basal exopher levels observed among wild-type controls are associated with the use of different transgenes for exopher visualization, as detailed in Supplementary Data 3. Data are presented as median with interquartile range. Statistical analyses were performed using the Kruskal-Wallis test with Dunn's multiple comparisons test; non-significant p values ($p > 0.05$) are in pink colour, significant p values ($p < 0.05$) are in blue colour.

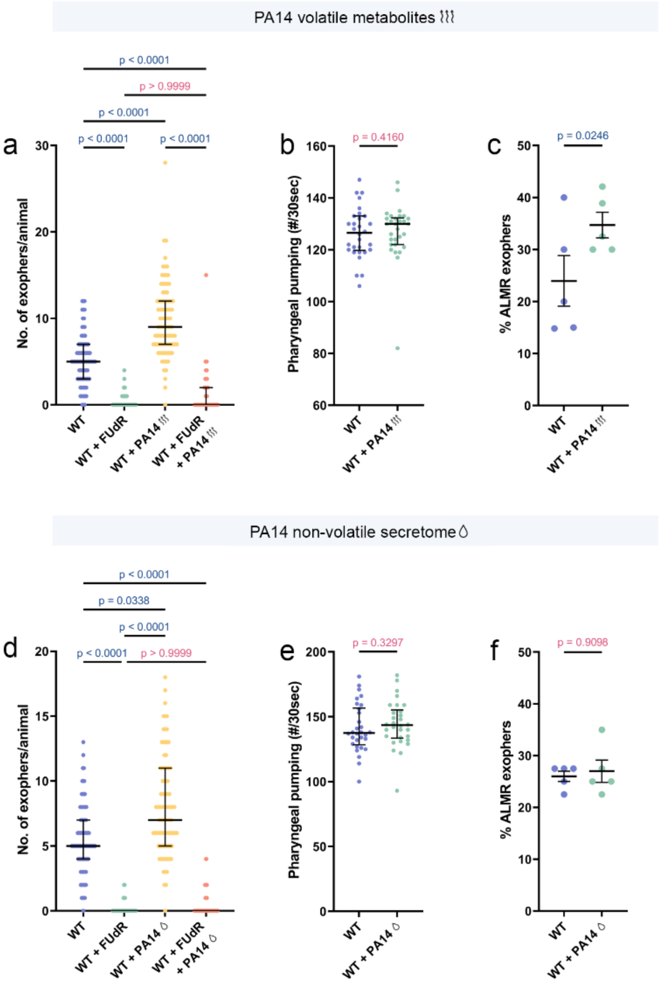

Supplementary Figure 2. PA14 volatile and non-volatile secretomes exposure differentially affects exopher production in neurons and muscles without altering feeding behavior.

a FUdR treatment significantly reduced muscular exopher production upon exposure to PA14 volatile metabolites (n = 91, 88, 91, and 87 worms (for respective columns), N = 3 independent experiments).

b Exposure to PA14 volatile metabolites does not affect worm feeding behavior (n = 30 worms (for each column), N = 3 independent experiments).

c ALMR neurons show increased exopher production after exposure to PA14 volatile metabolites (n = 142 and 147 worms (for respective columns), N = 5 independent experiments).

d FUdR treatment significantly reduced exopher production upon exposure to PA14 non-volatile secretome (n = 88, 88, 91, and 79 worms (for respective columns), N = 3 independent experiments).

e Exposure to PA14 non-volatile secretome does not affect worm feeding behavior (n = 30 worms (for each column), N = 3 independent experiments).

f PA14 non-volatile secretome has no effect on exopher release from ALMR neurons (n = 200 worms (for each column), N = 5 independent experiments).

Data information: Data are presented as medians with interquartile ranges. Statistical analyses were performed using the Kruskal-Wallis test with Dunn’s multiple comparisons test (a, d), the two-tailed Mann-Whitney test (b, e) and Fisher's exact test (c, f), with non-significant p-values ($p > 0.05$) in pink and significant p-values ($p < 0.05$) in blue.

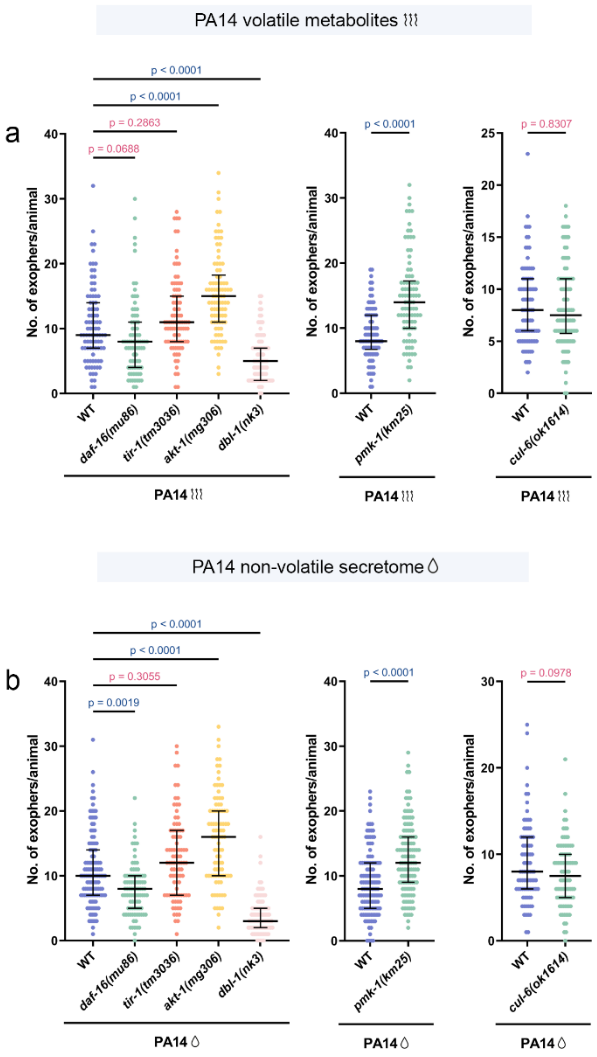

Supplementary Figure 3. Animals deficient in key immune signaling pathways exhibit altered exopher production in response to pathogenic bacterial metabolites.

a Exopher production in immune signaling mutants following exposure to PA14 volatile metabolites (n = 90, 80, 90, 90, and 90 worms (for respective columns), N = 3 independent experiments; n = 90 worms (for each column), N = 3 independent experiments; n = 87 and 90 worms (for respective columns), N = 3 independent experiments).

b Immune signaling mutants show altered exopher production in response to PA14 non-volatile secretome (n = 120, 90, 90, 90, and 90 worms (for respective columns), N = 4 independent experiments); n = 122 worms (for each column), N = 4 independent experiments; n = 85 and 90 worms (for respective columns), N = 3 independent experiments).

Data information: Differences in basal exopher levels observed among wild-type controls are associated with the use of different transgenes for exopher visualization, as detailed in Supplementary Data 3. Data are presented as median with interquartile range. Statistical analyses were performed using the Kruskal-Wallis test with Dunn's multiple comparisons test (a, b left panel) and the two-tailed Mann–Whitney test (a, b middle and right panels); non-significant p values ($p > 0.05$) are in pink colour, significant p values ($p < 0.05$) are in blue colour.

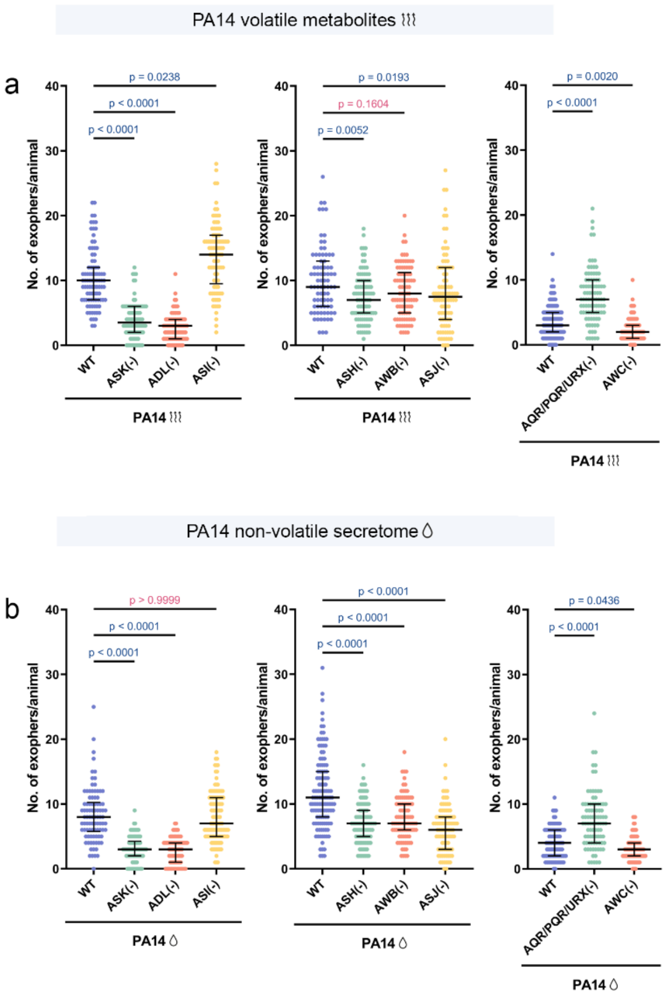

Supplementary Figure 4. Neuronal ablation of sensory neurons alters exopher production in response to PA14 metabolites.

a Exopher production in neuronal ablation strains upon exposure to PA14 volatile metabolites (n = 90, 88, 88, and 89 worms (for respective columns), N = 3 independent experiments, n = 80, 90, 90, and 90 worms (for respective columns), N = 3 independent experiments; n = 120, 90, and 90 worms (for respective columns), N = 3-4 independent experiments).

b Exopher production in neuronal ablation strains upon exposure to PA14 non-volatile secretome (n = 90, 90, 90, and 120 worms (for respective columns), N = 3-4 independent

experiments; n = 120, 90, 90, and 90 worms (for respective columns), N = 3-4 independent experiments; n = 90 worms (for each column), N = 3 independent experiments).

Data information: Differences in basal exopher levels observed among wild-type controls are associated with the use of different transgenes for exopher visualization, as detailed in Supplementary Data 3. Data are presented as median with interquartile range. Statistical analyses were performed using the Kruskal-Wallis test with Dunn's multiple comparisons test; non-significant p values ($p > 0.05$) are in pink colour, significant p values ($p < 0.05$) are in blue colour.

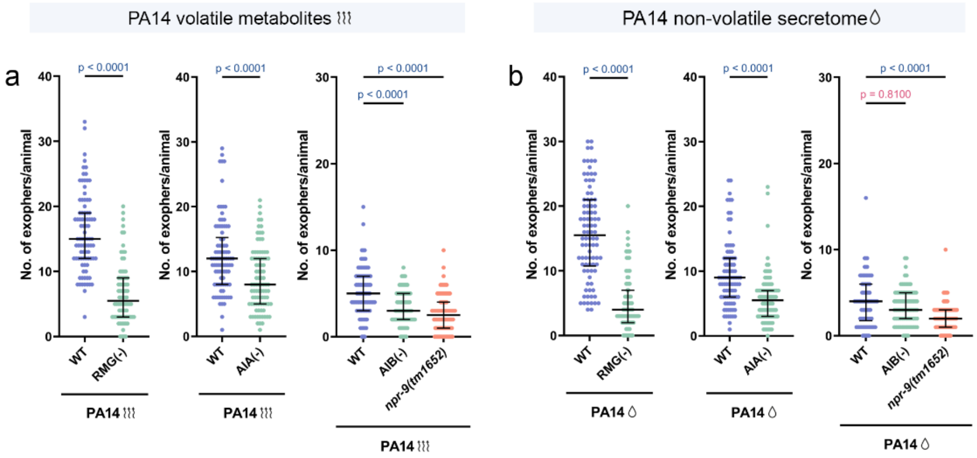

Supplementary Figure 5. Exopher production in response to PA14 volatile and non-volatile secretomes is decreased after removal of AIB, AIA and RMG interneurons and NPR-9 receptor.

a Animals lacking AIB, AIA, or RMG interneurons, as well as the NPR-9 receptor, produce fewer exophers following exposure to PA14-derived volatile metabolites (n = 88 and 78 worms (for respective columns), N = 3 independent experiments; n = 90 worms (for each column), N = 3 independent experiments; n = 90, 88, and 90 worms (for respective columns), N = 3 independent experiments).

b Animals lacking AIA or RMG interneurons, as well as the NPR-9 receptor, produce fewer exophers following exposure to PA14 non-volatile secretome, whereas AIB-ablated animals show no significant change (n = 90 worms (for each column), N = 3 independent experiments; n = 90 worms (for each column), N = 3 independent experiments; n = 90 worms (for each column), N = 3 independent experiments).

Data information: Differences in basal exopher levels observed among wild-type controls are associated with the use of different transgenes for exopher visualization, as detailed in Supplementary Data 3. Data are presented as median with interquartile range. Statistical analyses were performed using the Kruskal-Wallis test with Dunn's multiple comparisons test

and the two-tailed Mann–Whitney test; non-significant p values ($p > 0.05$) are in pink colour, significant p values ($p < 0.05$) are in blue colour.

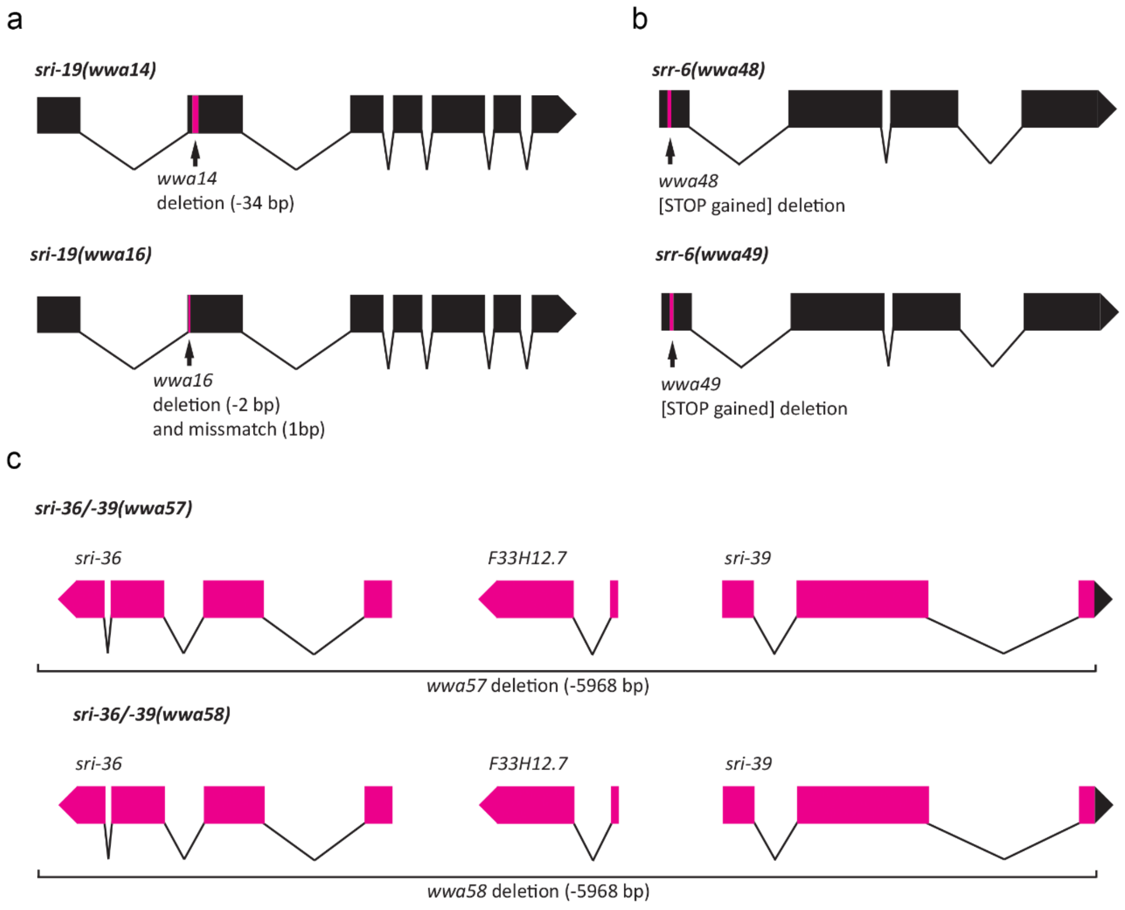

Supplementary Figure 6. Diagrams of (a) *sri-19*, (b) *srr-6*, (c) *sri-36*, and *sri-39* genomic structures and genetic lesions (marked in pink) used in the study (related to Materials and Methods).

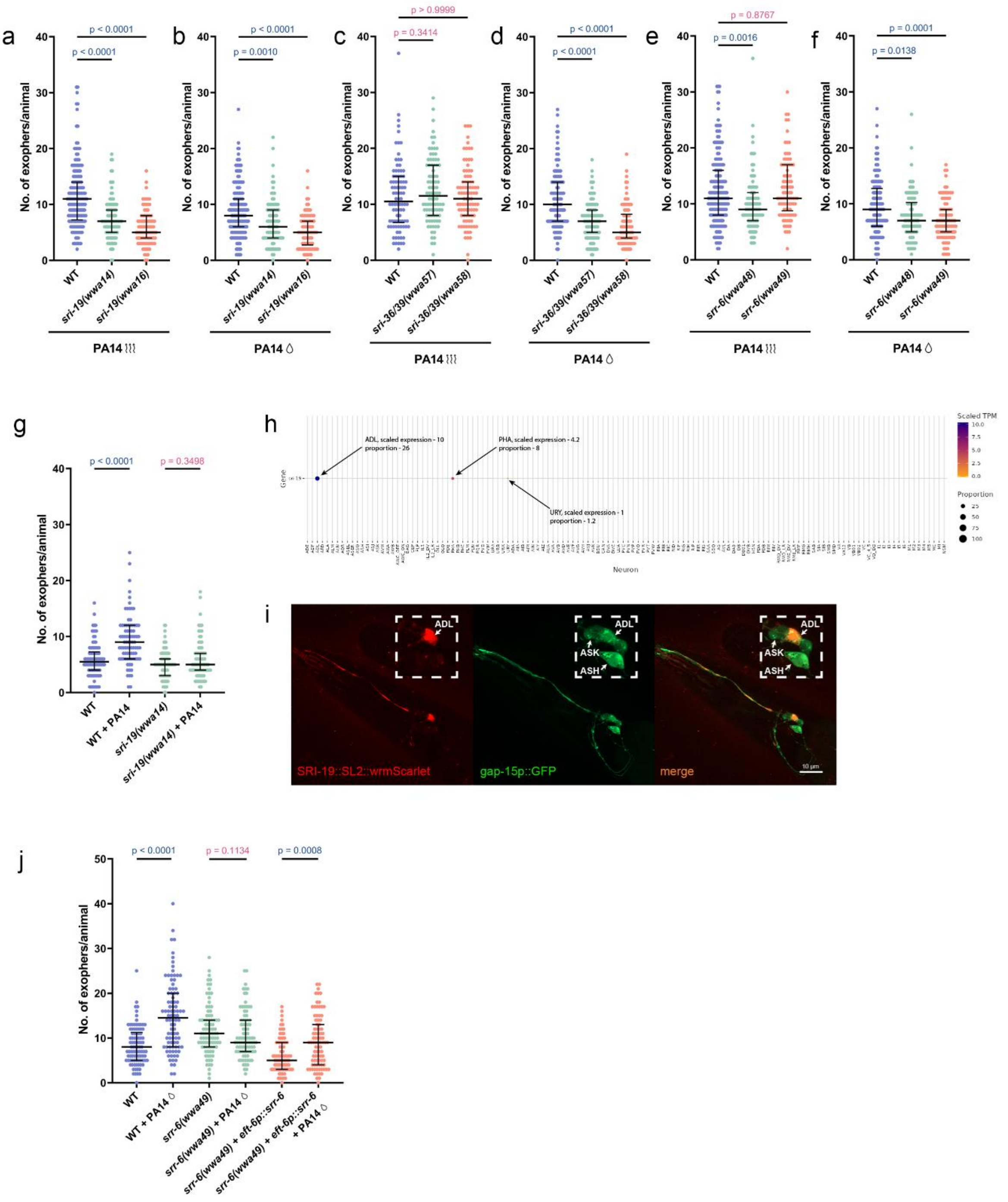

Supplementary Figure 7. Deletion of SRI-19, SRI-36, SRI-39, and SRR-6 receptors modifies exopher release triggered by PA14-produced secretomes.

a-b *sri-19* deletion alters exopher production in response to PA14 volatile and non-volatile secretomes (n = 180, 108, and 90 worms (for respective columns), N = 3 independent

experiments; n = 180, 90, and 90 worms (for respective columns), N = 3 independent experiments).

c-d *sri-36/39* deletion alters exopher production in response to PA14 non-volatile secretome but not to volatile metabolites (n = 90 worms (for each column), N = 3 independent experiments; n = 120, 120, and 90 worms (for respective columns), N = 3-4 independent experiments).

e-f *srr-6* deletion alters exopher production in response to PA14 non-volatile secretome but not to volatile metabolites (n = 180, 90, and 90 worms (for respective columns), N = 3 independent experiments; n = 180, 90, and 90 worms (for respective columns), N = 3 independent experiments).

g SRI-19 receptor is required for exopher release during PA14 infection (n = 90, 84, 90, and 75 worms (for respective columns), N = 3 independent experiments).

h Single-cell RNA-seq data from CeNGENApp[1] – *sri-19* is expressed mostly in ADL sensory neurons. Expression also occurs in PHA and URY neurons. The circle diameter represents the proportion of neurons in each cluster that express the sri-19 gene.

i Expression of SRI-19 receptor co-localizes with *gap-15* GFP-based reporter line in ADL neurons, consistent in at least 20 animals from three replicates. Scale bar is 10 μm.

j Expression of *srr-6* under intestine-specific promoter rescues exopher production in *srr-6* loss-of-function mutants (n = 94, 90, 90, 90, 80, and 87 worms (for respective columns), N = 3 independent experiments).

Data information: Data are presented as median with interquartile range. Statistical analyses were performed using the Kruskal-Wallis test with Dunn's multiple comparisons test (a-f) and the two-tailed Mann–Whitney test (g,j); non-significant p values ($p > 0.05$) are in pink colour, significant p values ($p < 0.05$) are in blue colour.

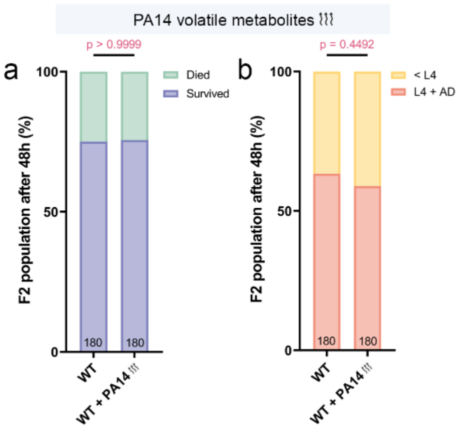

Supplementary Figure 8. Exposure of P0 hermaphrodites to PA14 volatile metabolites had no significant impact on F2 survival (a) and development (b) during subsequent PA14 infection.

Data information: (a) n = 180 worms (for each column), N = 3 independent experiments, (b) n = 180 worms (for each column), N = 3 independent experiments. Statistical analyses were performed using the Fisher's exact test; non-significant p values ($p > 0.05$) are in pink colour.

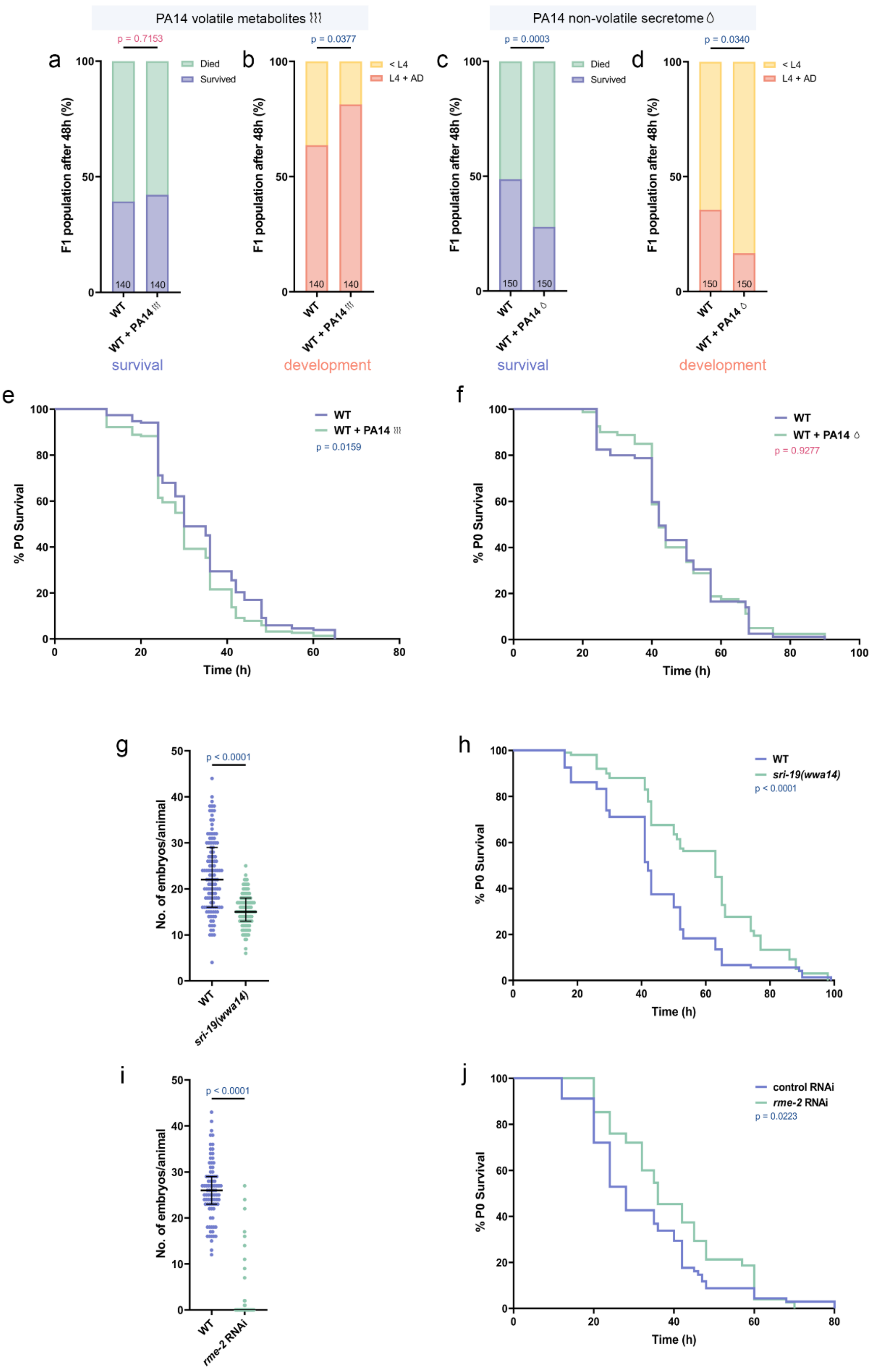
PA14 volatile metabolites
PA14 non-volatile secretome
a
b
c
d
p = 0.7153
p = 0.0377
p = 0.0003
p = 0.0340
Died
Survived
< L4
L4 + AD
F1 population after 48h (%)
WT
WT + PA14
survival
development
e
f
% P0 Survival
Time (h)
p = 0.0159
p = 0.9277
g
h
No. of embryos/animal
p < 0.0001
sri-19(wwa14)
i
j
control RNAi
rme-2 RNAi
p = 0.0223

Supplementary Figure 9. F1 development at 25°C is accelerated by P0 pre-exposure to volatile metabolites and impaired by non-volatile secretome.

a F1 progeny of wild-type worms exposed to PA14 volatile metabolites show no significant difference in survival after 48 hours of PA14 infection compared to the control group (n = 140 worms (for each column), N = 3 independent experiments).

b Parental exposure to PA14-derived volatile metabolites significantly improves developmental progression of the F1 generation after 48 hours on PA14 compared to the control group (n = 140 worms (for each column), N = 3 independent experiments).

c Parental exposure to PA14 non-volatile secretome significantly reduces the survival of F1 progeny upon PA14 infection compared to the control group (n = 150 worms (for each column), N = 3 independent experiments).

d F1 progeny of parents exposed to PA14 non-volatile secretome show reduced developmental progression during PA14 infection compared to the control group (n = 150 worms (for each column), N = 3 independent experiments).

e Pre-exposure to PA14 volatile metabolites reduces survival of P0 worms during subsequent PA14 infection (n = 153 worms (for each group), N = 6 independent experiments).

f Pre-exposure to PA14 non-volatile secretome does not significantly affect survival of P0 generation during PA14 infection (n = 79 and 80 worms (for respective columns), N = 3 independent experiments).

g Loss of SRI-19 reduces embryo accumulation (n = 119 worms (for each column), N = 4 independent experiments).

h The SRI-19 receptor that regulate exopher production upon exposure to PA14 non-volatile metabolites also regulates survival in response to PA14 infection (n = 104 and 98 worms (for respective groups), N = 4 independent experiments).

i *rme-2* knockdown lowers embryo accumulation (n = 88 and 89 worms (for respective columns), N = 3 independent experiments).

j Worms with *rme-2* gene knockdown, which produce low number of exophers, demonstrated enhanced survival rates upon PA14 infection (n = 68 and 75 worms (for respective groups), N = 4 independent experiments).

Data information: Experiments conducted at 25°C. Data are presented as stacked bar plots (a-d) and Kaplan–Meier survival curves (e, f, h, j); Statistical analyses were performed using the Fisher's exact test (a-d), long-rank (Mantel-Cox) test (e, f, h, j), and the two-tailed Mann–Whitney test (g, i); non-significant p values ($p > 0.05$) are in pink colour, significant p values ($p < 0.05$) are in blue colour.

References

1. Taylor, S. R. *et al.* Molecular topography of an entire nervous system. *Cell* **184**, 4329-4347.e23 (2021).
