# Supplementary material for: Volatile and non-volatile pathogen cues shape host extracellular vesicles production in pre-infection response
Source: Nat Commun. 2025 Dec 21;17:1038. doi: 10.1038/s41467-025-67789-z (PMC12847967; doi:10.1038/s41467-025-67789-z)
Supplement: Supplementary file 2 — Description of Additional Supplementary Information [file 41467_2025_67789_MOESM2_ESM.pdf]

Description of Additional Supplementary Files

File Name: Supplementary Data 1
Description: LC–MS identification of differentially abundant metabolites in exopher-inducing and non-inducing filtrates.

File Name: Supplementary Data 2
Description: Differential gene expression between high- and low-exopher-producing worms.

File Name: Supplementary Data 3
Description: List of Caenorhabditis elegans strains used in this study.

File Name: Supplementary Data 4
Description: List of materials, oligonucleotides, and plasmids used in this study.
